# Repurposing the Trypanosomatidic GSK Kinetobox for the Inhibition of Parasitic Pteridine and Dihydrofolate Reductases

**DOI:** 10.3390/ph14121246

**Published:** 2021-11-30

**Authors:** Matteo Santucci, Rosaria Luciani, Eleonora Gianquinto, Cecilia Pozzi, Flavio di Pisa, Lucia dello Iacono, Giacomo Landi, Lorenzo Tagliazucchi, Stefano Mangani, Francesca Spyrakis, Maria Paola Costi

**Affiliations:** 1 Department of Life Science, University of Modena and Reggio Emilia, Via Campi 103, 41125 Modena, Italy; matteo.santucci86@gmail.com (M.S.); rosaria.luciani@libero.it (R.L.); lorenzo.tagliazucchi@unimore.it (L.T.); 2 Department of Drug Science and Technology, University of Turin, Via Giuria 9, 10125 Turin, Italy; eleonora.gianquinto@unito.it (E.G.); francesca.spyrakis@unito.it (F.S.); 3 Department of Biotechnology, Chemistry and Pharmacy—Department of Excellence 2018–2020, University of Siena, Via Aldo Moro 2, 53100 Siena, Italy; pozzi4@unisi.it (C.P.); dipisa2@unisi.it (F.d.P.); delloiacono3@unisi.it (L.d.I.); landi31@unisi.it (G.L.); stefano.mangani@unisi.it (S.M.)

**Keywords:** GSK Kinetobox, PTR1, DHFR-TS, Leishmaniasis, trypanosomiasis, drug discovery, molecular modelling, medium throughput screening

## Abstract

Three open-source anti-kinetoplastid chemical boxes derived from a whole-cell phenotypic screening by GlaxoSmithKline (Tres Cantos Anti-Kinetoplastid Screening, TCAKS) were exploited for the discovery of a novel core structure inspiring new treatments of parasitic diseases targeting the trypansosmatidic pteridine reductase 1 (PTR1) and dihydrofolate reductase (DHFR) enzymes. In total, 592 compounds were tested through medium-throughput screening assays. A subset of 14 compounds successfully inhibited the enzyme activity in the low micromolar range of at least one of the enzymes from both *Trypanosoma brucei* and *Lesihmania major* parasites (pan-inhibitors), or from both PTR1 and DHFR-TS of the same parasite (dual inhibitors). Molecular docking studies of the protein–ligand interaction focused on new scaffolds not reproducing the well-known antifolate core clearly explaining the experimental data. TCMDC-143249, classified as a benzenesulfonamide derivative by the QikProp descriptor tool, showed selective inhibition of PTR1 and growth inhibition of the kinetoplastid parasites in the 5 μM range. In our work, we enlarged the biological profile of the GSK Kinetobox and identified new core structures inhibiting selectively PTR1, effective against the kinetoplastid infectious protozoans. In perspective, we foresee the development of selective PTR1 and DHFR inhibitors for studies of drug combinations.

## 1. Introduction

Neglected tropical diseases (NTDs) are a diverse set of 20 diseases that cause a devastating human, social and economic burden on more than 1 billion people worldwide, predominantly in tropical and subtropical areas [1]. Trypanosomatids are single-celled protozoan parasites, which cause various diseases such as Leishmaniasis, Chagas disease and human African trypanosomiasis (HAT), all known as vector borne parasitic diseases [2,3]. The little or no prospects of financial gain has made the pharmaceutical industry show low interest in developing new drugs for NTDs [4]. The treatment with currently available drugs, discovered decades ago, presents many drawbacks, such as high toxicity, poor efficacy, difficulties in administration and drug resistance [5,6,7,8,9]. Thus, there is an urgent need to discover new, improved and affordable drugs as well as promising drug targets for the design of new antiparasitic compounds.

To this end, the enzymes belonging to the folate pathway, pteridine reductase 1 (PTR1) and dihydrofolate reductase-thymidylate synthase (DHFR-TS), represent interesting targets [10,11,12]. PTR1 is a short-chain dehydrogenase/reductase (SDR), involved in the biosynthesis of reduced folate, a housekeeping cofactor for the synthesis of 2′-deoxythymidine-5′-monophosphate (dTMP) needed for DNA synthesis [13,14]. PTR1 is responsible for the main resistance mechanism to the treatment with antifolate drugs targeting bifunctional DHFR-TS in infections caused by *Leishmania* and *Trypanosoma* parasites [15,16]. Indeed, given its ability of reducing folates, PTR1 acts as a metabolic bypass when DHFR-TS is inhibited [17]. Under these conditions, PTR1 expression levels highly increase, and this can guarantee the production of 10% of tetrahydrofolate required by the cell to sustain the parasite survival [18]. An effective treatment of trypanosomatid infections could be achieved through the simultaneous inhibition of DHFR-TS and PTR1 by a single drug or a combination of compounds that are specific and selective inhibitors of each target [19].

We have previously reported the identification of PTR1-specific inhibitors and used them in combination with known DHFR-TS inhibitors to improve the in vitro efficacy against *Leishmania* and *Trypanosoma* species, and to reduce the treatment toxicity with respect to administering DHFR-TS inhibitors alone [20]. Among the many available compound libraries that can be used for screening purposes against relevant target proteins, the Kinetobox [21], provided as open resource by GlaxoSmithKline company, is still unexplored against the folate dependent enzymes. The library was largely evaluated against several different microorganisms and targets, such as *Crithidia fasciculata*, a non-mammalian infective lower trypanosomatid [22]; glycogen synthase kinase-3 (GSK-3) as a druggable target for the human protozoan parasite *Leishmania* [23]; and cruzipain enzyme, a sulfated glycoprotein acting as main cysteine peptidase of *Trypanosoma cruzi*, playing an important role in Chagas disease [24]. The screening of the same compound library against many different target organisms and proteins involved in several diseases can be conceptually linked to the repurposing approach and can lead to the identification of novel chemical structures (core structures) against the selected targets [25,26].

In this work, we sought to exploit three anti-kinetoplastid chemical boxes (Kinetoboxes, namely LEISH-, CHAGAS- and HAT-box) for the identification of new selective inhibitors of PTR1 showing core structures different from the known folate/pyrimidine ones [21]. Each Kinetobox was clustered from the original GSK collection including 1.8 million compounds, according to whole-cell assays performed for the three kinetoplastids (*Leishmania donovani*, *T. cruzi* and *Trypanosoma brucei*). A total of 592 compounds have been identified as the most active: 192 were active against *L. donovani* (LEISH-box), 222 against *T. cruzi* (CHAGAS-box) and 192 against *T. brucei* (HAT-box). Interestingly, 88% of the selected chemical collection was not previously published and the remaining 12% showed an activity profile unrelated to the activity against *Leishmania* or *Trypanosoma.* Moreover, the three boxes did not contain structural analogs of drugs currently used in the clinic for Leishmaniasis, Chagas disease or HAT. To identify inhibitors of *Leishmania major* (*Lm*) and *T. brucei* (*Tb*) PTR1, we firstly analyzed the chemical–structural features of the compound library and then performed a medium-throughput screening (MTS) assay against *Lm* and *Tb* forms of DHFR-TS and PTR1 enzymes. Some active compounds having an antifolate scaffold were identified, with some of them showing a pan-inhibitor character, when they inhibit PTR1 from both *Lm* ad *Tb* kinetoplastids, and others showing dual inhibitors, when they inhibit both PTR1 and DHFR-TS of at least one parasitic species. Interestingly, some novel structures different from the folate core were also identified.

Compound **TCMDC-143249**, a benzenesulfonamide derivative, having an in vivo efficacy towards both parasites in the low micromolar range, was able to selectively inhibit in vitro both *Lm*PTR1 and *Tb*PTR1, but not the corresponding DHFR-TSs. Molecular modelling studies showed that the inhibitor mimics the substrate pose in both *Tb*PTR1 and *Lm*PTR1, while it does not fulfill active site binding requirements in *Tb*DHFR-TS nor in *Lm*DHFR-TS, hence providing a structural basis for the differential activity of **TCMDC-143249** compound in PTR1 and DHFR-TS. The homology model of *Lm*DHFR-TS was obtained through computational studies for docking purposes. The present study also proposes a novel core structure that can be exploited for the development of new anti-parasitic compounds.

## 2. Results and Discussion

### 2.1. In Silico Evaluation of Drug-Likeness Properties and Hierarchical Clustering Analysis

**Figure 1:**
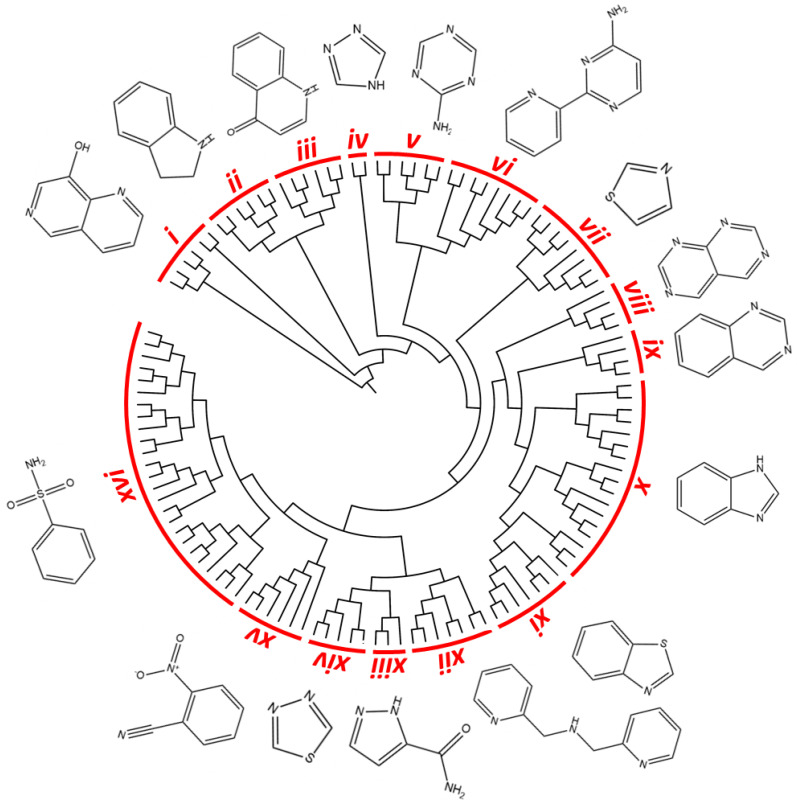
Similarity-based hierarchical clustering analysis. The figure shows the dendrogram of the Kinetobox compound library. Clustering analysis was based on the chemical similarity. Sixteen clusters were identified as the most representative of the entire compound collection; the chemical core structure of the cluster compounds is shown.

A chemoinformatic in silico analysis was firstly performed to characterize the drug-likeness properties and chemical space of the Kinetobox collection, aiming to identify its most representative chemical core structure using the QikProp descriptor tool (Canvas software-Schrödinger) [27,28]. For each compound, molecular weight (MW), alogP, number of H-bond acceptors (HBA) and H-bond donors (HBD), total polar surface area (TPSA) and number of rotatable bonds have been analyzed (Table 1). Assuming no more than one violation of the rule [29], 92.2% of the entire library was in accordance with Lipinski’s “rule of five (RO5)” [30]. The MW of the compounds ranged from 210 g/mol to 547 g/mol. The calculated lipophilicity (alogP) ranged from −2.70 to 5.84. The number of hydrogen bond acceptors (HBAs) was 1–8, and the number of hydrogen bond donors (HBDs) varied from 0 to 5. Extending the RO5 evaluation to include properties associated with favorable bioavailability [31], the library showed a total polar surface area (TPSA) in the range 19 Å^2^–184 Å^2^, and between 1 and 11 rotatable bonds, with a mean value of 5.1. The chemical space of the entire Kinetobox library was also properly explored. A similarity-based hierarchical clustering analysis was performed to identify its most representative chemical clusters. Initially, 119 compound clusters were identified based on functional type and hybridization grade of atoms and bonds. The single core structures populating each cluster were further visually inspected and clusters sharing the same chemical core structure were grouped together. In this way, it was possible to reduce to 16 the number of clusters covering the total chemical space of the Kinetobox collection. The most representative are: *i*—1,6-naphthyridin-8-ol derivatives; *ii*—indoline-derivatives; *iii*—3yridine3-4(1H)-one derivatives; *iv*—4H-1,2,4-triazoles; *v*—1,3,5-triazin-2-amines; *vi*—2-(3yridine-2-yl)pyrimidin-4-amines; *vii*—thiazoles; *viii*—pyrimido[4,5-d]pyrimidines; *ix*—quinazolines; *x*—imidazole and 1H-benzo [d]imidazole derivatives; *xi*—benzo [d]thiazoles; *xii*—bis(3yridine-2-ylmethyl)amines; *xiii*—1H-pyrazole-5-carboxamides; *xiv*—1,3,4 thiadiazole derivatives; *xv*—2-nitrobenzonitrile derivatives; *xvi*—benzenesulfonamides (Figure 1). Interestingly, not all the core structure were known hits or leads in the field of anti-trypanosomatidic folate enzyme inhibitors, and therefore, we confirmed our interest in the on-target MTS study.

### 2.2. Inhibition of PTR1s and DHFRs

**Figure 2:**
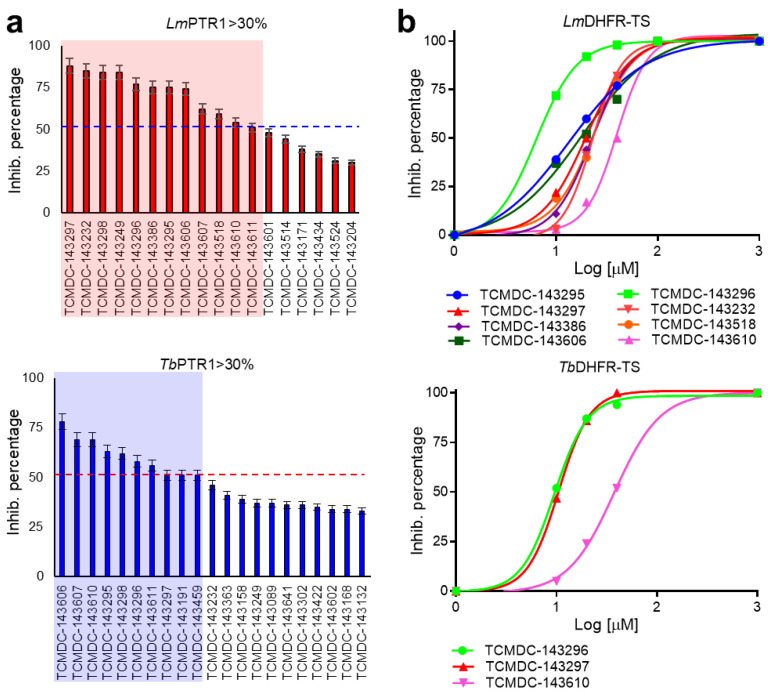
First in vitro screening assay on *Lm/Tb*PTR1 and *Lm/Tb*DHFR-TS, and IC_50_ evaluation. (**a**) The percentage values of inhibition of the compounds inhibiting PTR1 enzymes with an efficacy cut-off value ≥ 50% at 10 μM (red and blue square for *Lm* and *Tb*PTR1, respectively). Among these, a subset of 14 compounds, including 10 pan-inhibitors and 4 additional compounds inhibiting the recombinant protein of one single parasitic agent, was selected as starting point for the secondary screening on *Lm/Tb*DHFR-TS. (**b**) The resulting four-parameter Hill dose–response curve of the most potent compounds active on DHFR-TS protein from *L. major* and *T. brucei*. Only 3 compounds showed inhibition efficacy for *Tb*DHFR-TS in a medium-high micromolar range (9.7–38.2 µM); 8 compounds showed IC_50_ values in 6.9–40.0 µM range against *Lm*DHFR-TS.

**Figure 3:**
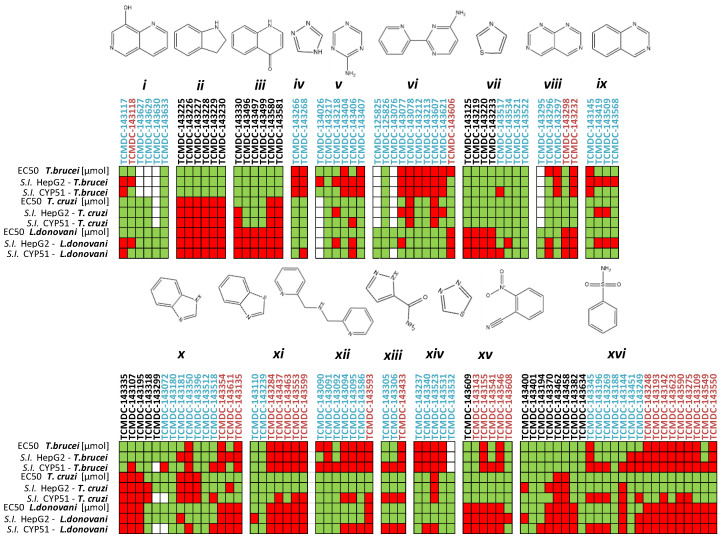
Inhibition growth rate and early toxicological data of the compounds belonging to the 16 clusters identified by similarity-based analysis as the most representative of the entire Kinetobox. The data ranges are reported using a traffic light system. EC_50_ values for *T. brucei*, *L. major* and *T. cruzi* and selectivity index (*S.I.*) with respect to CYP450 and HepG2 cells are reported. The cells are green colored when the EC50 vs. *Tc/Ld/Tb* parasites is >10 µM, *S.I.* HepG2 and *S.I.* CYP51 ≥ 10, and red when data indicate no activity and toxicity (EC_50_ vs. *Tc/Ld/Tb* proteins ≥ 10 µM, *S.I.* HepG2 and CYP51 > 10). * White corresponds to “no data available”. Compound labels: black, HAT-box compounds (*T. brucei*); cyan, LEISH-box compounds (*L. donovani*); magenta, CHAGAS-box compounds (*T. cruzi*).

The capability of the Kinetobox to inhibit the enzyme activity was tested in vitro at 10 µM against PTR1 recombinant protein from *T. brucei* and *L. major*, by a medium-high throughput screening assay. The inhibition percentages of each compound were determined, and the corresponding IC_50_ values were evaluated in a secondary screening only for the most active molecules (Table 2, Table 3 and Table 4). We ranked the total compounds according to the inhibition results, focusing on those showing a cut-off value ≥ 50% for *Lm*PTR1 or *Tb*PTR1. In this way, 10 and 12 molecules, corresponding to a success rate ≤ 2%, were selected to inhibit *Tb*PTR1 and *Lm*PTR1 in the range 6.4–13.5 µM and 5.7–9.8 µM, respectively (Figure 2a). To select the compounds that can inhibit PTR1 from both parasitic species (pan-inhibitors), a shortlist of 10 molecules was selected and finally enriched with four additional molecules: **TCMDC-143191** and **TCMDC-143459** inhibiting *Tb*PTR1 with an inhibition percentage of 51% at 10 µM and an IC_50_ of 9.8 µM; **TCMDC-143386** and **TCMDC-143518** as selective inhibitors of *Lm*PTR1 showing percentages of inhibition of 75% and 59% at 10 µM and IC_50_ of 6.7 and 8.5 µM, respectively. The 14 compounds were further tested towards *Lm*/*Tb*DHFR-TS (secondary screening), to select molecules inhibiting both PTR1 and DHFR-TS enzymes of at least one kinetoplastid (dual inhibitors). Three compounds showed IC_50_ values for *Tb*DHFR-TS in the 9.7–38.2 µM range. Conversely, the same library was more active against *Lm*DHFR-TS, with eight compounds showing IC_50_ values between 6.9 µM and 40.0 µM (Figure 2b). Notably, only two pteridine-based compounds (**TCMDC-143296** and **TCMDC-143297**) belonging to the LEISH-box inhibited *Lm*/*Tb*PTR1 at 6.5–8.6 µM and 5.7–9.8 µM, respectively. We further investigated the relationship between in vitro potency and in vivo inhibition growth on parasite. These latest data were provided as associated data from the open resource GSK database (Table 2, Table 3 and Table 4) and were thus available for our studies. We firstly filtered, from the entire GSK dataset, the data relative to compounds populating the most representative clusters of the entire Kinetobox. Figure 3 reports a heat-map showing the in vivo anti-parasitic activity towards *Leishmania* and *Trypanosoma* parasites for all compounds of each single cluster and the early toxicological profile in terms of toxicity with respect to cytochrome P450 (CYP51) and human liver cancer cell line (HepG2). The compounds belonging to different kinetoplastid boxes but sharing the same chemical core structure show a similar anti-parasitic activity profile. Interestingly, compound **TCMDC-143249** (LEISH box) belongs to the cluster of benzenesulfonamide derivatives with IC_50_ of 6.0 µM for *Lm*PTR1 and shows *Leishmania* parasite inhibition growth with EC_50_ of 5.6 µM. The compound can also inhibit the growth rate of *T. brucei* and *T. cruzi* with EC_50_ values equal to 6.3 µM and 4.2 µM, respectively [21].

### 2.3. Molecular Docking

To investigate the inhibition mechanism of the 14 selected compounds, we performed molecular docking studies in *Tb*PTR1 and *Lm*PTR1, but also in *Tb*DHFR-TS and *Lm*DHFR-TS, paying particular attention to the binding mode of the different scaffolds (Table S1). The X-ray crystal structure of *Lm*DHFR-TS is not available, and for docking purposes, we built the 3D structure through comparative homology modelling. We chose as a template the structure of DHFR-TS from *T. cruzi* (PDB ID 3INV), given the high sequence identity of the isoforms (about 69%). The model was built through SWISS-Model and the corresponding Ramachandran plot was generated with Molprobity for assessing the model quality [32,33]. The NADPH cofactor was retained as reported in the template. As reported below, we found that the results obtained from the docking analysis of the 14 compounds against the *Lm*DHFR-TS model agree with the observed experimental data. These results explained on a structural basis how the inhibitor–enzyme interactions can support the inhibition effect of the enzyme, thus qualitatively validating our model.

In PTR1 and DHFR-TS, inhibitors may assume a substrate-like or an antifolate-like pose, depending on the hydrogen bond donor/acceptor pattern of interaction. We adopted two well-known human DHFR inhibitors and drugs in therapy, methotrexate (MTX) and pemetrexed (Figure S1), as antifolate-like reference compounds in the docking studies. The X-ray crystal structures of the complex DHFR-TS:MTX and *Tb*PTR1:MTX were available in the PDB (PDB ID 2C7V). The X-ray structures of pemetrexed *Tb*PTR1 (PDB ID 2X9G) were also included in the study. In PTR1, the overall pose of the inhibitors is guided by the presence of a hydrogen bond donor/positively charged center, but also by an acceptor (Figure S1a,b). This is required for a direct hydrogen bond/electrostatic interaction with the NADPH pyrophosphate, while an acceptor is essential for a hydrogen bond to Arg14 and a water-mediated interaction with NADPH pyrophosphate. In DHFR-TS, only one hydrogen bond donor or a positively charged center (Figure S1c,d) is required for interacting with an aspartate residue, guiding, again, the overall binding mode of the molecule in one of the two poses. Thus, the selected 14 compounds were further classified according to their core structure in antifolate-like scaffolds (Table 2 and Table 3) and non-antifolate-like scaffolds (Table 4), and the cluster number identified in the chemoinformatic analysis was included, where possible (Figure 3). Not all 14 compounds could be assigned to one of identified clusters. 

**Figure 4:**
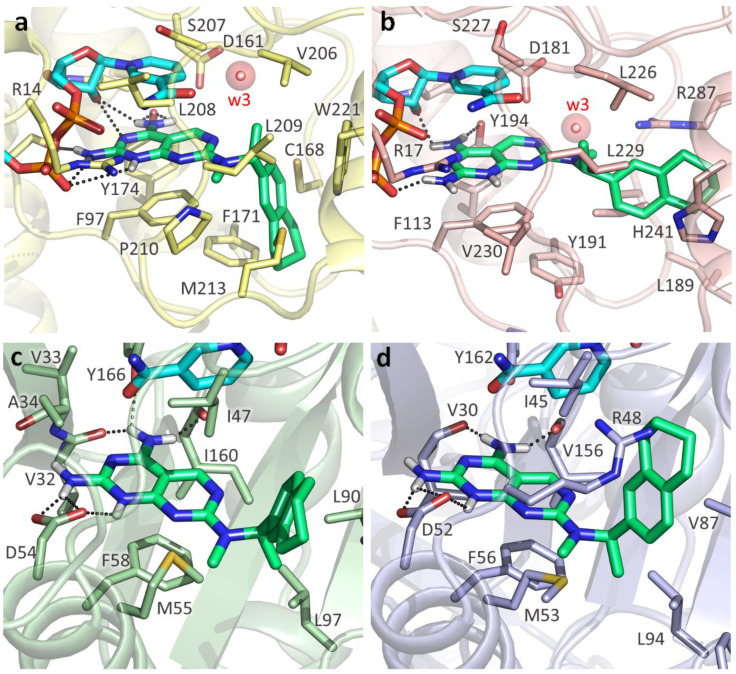
**TCMDC-143296** docking poses in the different reductases. (**a**) **TCMDC-143296** in *Tb*PTR1 (PDB ID:2C7V). (**b**) **TCMDC-143296** in *Lm*PTR1 (PDB ID:2BFA). (**c**) **TCMDC-143296** in *Tb*DHFR (PDB ID:3RG9). (**d**) **TCMDC-143296** in *Lm*DHFR-TS (model). Protein is represented as cartoon (*Tb*PTR1, light yellow; *Lm*PTR1, light pink; *Tb*DHFR, light green; *Lm*DHFR-TS, light blue). **TCMDC-143296** (green), NADPH cofactor (cyan) and binding site residues are depicted as sticks and labelled. Hydrogen bonds are indicated as black dashed lines. Water molecules, indicated when not displaced by the docking, are reported as red spheres with their Van der Waals radius visible in transparency.

Contrarily to antifolate-like scaffolds, whose binding pose is considered similar to the well-known antifolate methotrexate (MTX) and pemetrexed (Figure S1), the non-antifolate-like scaffolds display diverse features, and their binding mode could not be anticipated straightforwardly. Compounds from Table 2 and Table 4 were docked in *T. brucei* and *L. major* PTR1, as well as in DHFR-TS. From the molecular docking analysis, we observed that compounds from Table 2 and Table 3 bind both PTR1 and DHFR-TS with an antifolate-like pose. Overall, pyrimido-pyrimidine derivatives (Table 2) exerted low micromolar inhibition on both *Tb-* and *Lm*PTR1 enzymes, exhibiting no detectable anti DHFR-TS inhibition (IC_50_ > 40µM). **TCMDC-143296** (LEISH_BOX) showed a low EC_50_ against *T. brucei* and *L. donovani,* which might be linked to the dual low micromolar inhibition of PTR1 and DHFR-TS enzymes. Docking pose of **TCMDC-143296** illustrated that the pyrido-pyrimidine core traces pteridine interactions of MTX and other antifolates in both PTR1 and DHFR-TS, while the tetrahydronapthyl substituent occupies the region generally covered by the para-aminobenzoate moiety in MTX. In *Tb*PTR1, key H-bonds are formed with the catalytically important Tyr174, with the phosphate and the ribose of the cofactor, and a π–π sandwich is formed by the ligand pteridine moiety with Phe97 and the cofactor nicotinamide. As mentioned, the nitrogen in position 1 is protonated to favorably interact with the cofactor phosphate (Figure 4a). In *Lm*PTR1, H-bonds were maintained with the corresponding Tyr194 and with the cofactor phosphate and ribose (Figure 4b). With respect to the canonical antifolate pose (Figure 4a), the compound was slightly shifted, possibly because the substituent in position 7 might cause an opening of the binding site loop, which can hardly be accounted for in docking studies. However, as already observed [34], substituents in this position are generally better tolerated in *Lm*PTR1 than in *Tb*PTR1. When docked in *Tb*DHFR-TS, **TCMDC-143296** assumed an antifolate-like binding pose (Figure 4c), forming a π–π contact with Phe58, an electrostatic interaction with Asp54 and H-bonding to Val32, Ile47 and Tyr166 in *Tb*DHFR-TS (Figure 4c), and maintaining the corresponding contacts in *Lm*DHFR-TS (Figure 4d). Interestingly, the promising in vivo activity exerted towards *L. donovani* could be given by a synergic effect of the compound able to inhibit both PTR1 and DHFR-TS, as also observed for **TCMDC-143295** and **TCMDC-143297**. Other pyrimido-pyrimidine derivatives (namely **TCMDC-143232** and **TCMDC-143295**) inhibited *Tb*PTR1 and *Lm*PTR1 showing a similar pose in both enzymes, but they were not active against *Tb*DHFR-TS. Lastly, **TCMDC-143298** did not show any activity against both DHFR-TSs, and it demonstrated a promising EC_50_ on parasites.

Among the other compounds reported in Table 3, the pyrido-pyrimidine derivative **TCMDC-143606** showed an EC_50_ in the range of 20 μM in vitro towards both parasites, and IC_50_ in the range of 6 μM against *Tb*/*Lm*-PTR1. The docking pose in *Tb*PTR1 (Figure S2a) and *Lm*PTR1 is well conserved: the ligand forms an extended network of H-bonds with the cofactor, contacting the phosphates, the ribose, the nicotinamide and other residues lining the pocket as Asp161 and Tyr174. The π–π sandwich is conserved, and hydrophobic interactions are observed with Leu209 and Pro210. The most relevant interactions are maintained also in *Lm*DHFR-TS, where Val30, Asp52 and Val156 are H-bonded, a π–π and hydrophobic contacts are made with Phe56, Met53 and Val156, respectively (Figure S2b). **TCMDC-143607** showed good inhibition only towards *Tb*PTR1 and *Lm*PTR1, but an interesting in vivo activity was measured also for *T. brucei* and *L. donovani*. The pyrrolo-pyrimidine derivative **TCMDC-143610** and the pyrimidine derivative **TCMDC-143611** presented a similar docking pose in both *Tb*PTR1 and *Lm*PTR1, likely justifying the comparable in vitro inhibition activity. They are, in fact, both able to H-bond Tyr174 and the cofactor, while maintaining the π–π sandwich with Phe97 and the nicotinamide and forming hydrophobic contacts with Cys168, Phe171 and Leu209 (Figure S2c,d).

**Figure 5:**
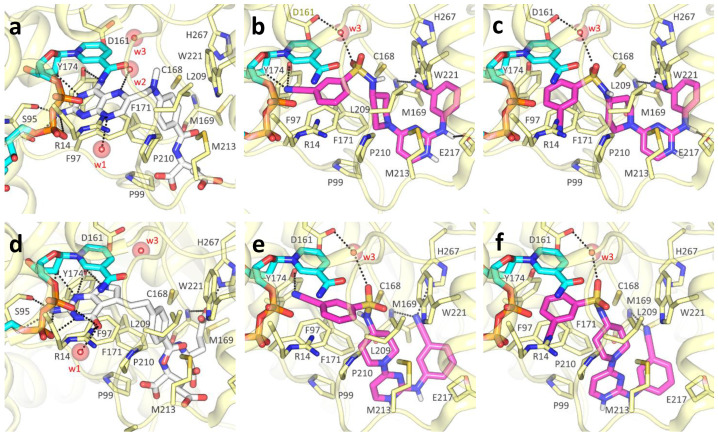
**TCMDC-143249** docking poses in TbPTR1. (**a**) MTX (white) main polar contacts in PDB ID 2C7V. (**b**,**c**) Two main orientations of **TCMDC-143249** (magenta) docked in PDB ID 2C7V. (**d**) Pemetrexed (white) main polar contacts in PDB ID 2X9G. (**e**,**f**) Two main orientations of **TCMDC-143249** (magenta) docked in PDB ID 2X9G. Protein is represented as light yellow cartoon, with relevant binding site residues depicted as sticks and labelled. NADPH cofactor (cyan) and ligands are shown as capped sticks; polar interactions between ligands and protein are shown as black dashed lines. Water molecules, indicated when not displaced by the docking, are reported as red spheres with their Van der Waals radius visible in transparency. For clarity, polar hydrogens are shown for ligands only.

**Figure 6:**
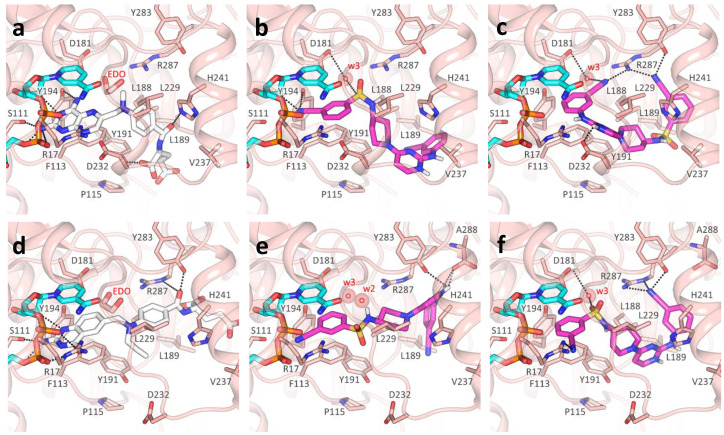
**TCMDC-143249** docking poses in LmPTR1. (**a**) MTX (white) main polar contacts in PDB ID 1E7W. (**b**,**c**) Two orientations of **TCMDC-143249** (magenta) docked in PDB ID 1E7W. (**d**) Co-crystallized dideazafolate ligand (white) main polar contacts in PDB ID 2BFA. (**e**,**f**) Two orientations of **TCMDC-143249** (magenta) docked in PDB ID 2BFA. Protein is represented as pink cartoon, with relevant binding site residues depicted as sticks and labelled. NADPH cofactor (cyan) and ligands are shown as capped sticks, polar interactions between ligands and protein are shown as black dashed lines. Water molecules, indicated when not displaced by the docking, are reported as red spheres with their Van der Waals radius visible in transparency. For clarity, polar hydrogens are shown for ligands only. 1,2-Ethanediol (EDO), a cryoprotectant mimicking ordered water molecules in PDB IDs 1E7W and 2BFA, is reported as sticks in a and d, and was substituted by water molecules w2 and w3 in docking studies (**b**,**c** and **e**,**f**).

Among non-pteridine compounds (Table 4), only **TCMDC-143249** inhibited both *Tb*PTR1 and *Lm*PTR1 in vitro within a low micromolar range. **TCMDC-143249** also showed a promising inhibition profile against both *T. brucei* and *L. donovani* in vivo, while being inactive against DHFR-TSs. As the H-bond donor/acceptor pattern in **TCMDC-143249** could not be associated either with antifolates or substrates, we deeply investigated its binding mode in our docking studies, as reported hereafter. The best docking results were achieved in *Tb*PTR1 structures complexed with MTX (Figure 5a) and pemetrexed (Figure 5d). These *Tb*PTR1 structures were chosen as reference for checking whether **TCMDC-143249** assumed an antifolate-like (as MTX) or substrate-like (pemetrexed) pose in the enzyme, engaging catalytically important residues such as Tyr174, Arg14 and ribose and phosphates of the NADPH cofactor [14]. In this framework, water molecules play a relevant role, bridging the ligand to Asp161 and to the NADPH pyrophosphate (w1, w2, w3 in Figure 5), and were thus retained in docking studies as reported in Table S1. The interaction and/or replacement of these water molecules may help the stabilization of the ligand binding pose. The two most favorable poses of **TCMDC-143249** in 2C7V (Figure 5b,c) and in 2X9G (Figure 5e,f) are reported and compared to MTX and pemetrexed binding poses (Figure 5a,d). Both orientations trace main interactions of the cognate ligands, but if the first pose resembles that of antifolate drugs (Figure 5b,e), the second is more similar to the substrate-like one (Figure 5c,f). In both cases, the 3-cyanophenyl moiety retains a π–π interaction with the nicotinamide ring of NADPH cofactor and Phe97. In the antifolate-like orientation, the nitrile substituent occupies a main H-bond acceptor site of pteridine ligands, contacting the ribose hydroxyl group and Tyr174. In the substrate-like orientation, the nitrile group H-bonds an Arg14 side chain and possibly displaces a water molecule (w1 in Figure S1a) occupying a crucial acceptor site for substrate anchoring. The sulfonamide moiety of **TCMDC-143249** may displace another water molecule (w2 in Figure 5a) in both orientations, interacting with Asp161 through a bridging water molecule (w3 in Figure 6a,c). Relevant hydrophobic interactions involve the piperidinyl ring of the ligand and residues Phe97, Phe171, Pro210 and Trp221. Lastly, moiety points toward the bulk, H-bonding Met169 and His267, while an ionic interaction the cyanophenyl-amino-pyrimidine may take place depending on the Glu217 side chain orientation and the protonation state of **TCMDC-143249**.

Moving to *Lm*PTR1, we can observe that co-crystallized ligands (Figure 6a,d) show the same rich network of polar contacts between the pteridine ring and residues Arg17, Ser111, Tyr194 and the NADPH cofactor. In PDB ID 1E7W (Figure 6a), the glutamate tail of MTX binds in a subsite lined by Leu188, Leu189, Leu229 and Asp232, H- bonding to Tyr191 and His241. In contrast to Trp221 in *Tb*PTR1, His241 in *Lm*PTR1 leaves room for a second subsite, flanked by the side chains of Tyr283, and by Arg287 and Ala288 belonging to the C-terminus of the adjacent protomer. This subsite can also be occupied by inhibitors, as shown by the ligand binding pose in PDB ID 2BFA.

Similarly to what was reported for *Tb*PTR1, the 3-cyanophenyl moiety of **TCMDC-143249** mimics the pteridine ring π–π sandwich interaction with Phe113 and the nicotinamide ring, contacting the NADPH and catalytically important residues such as Arg17, Asp181 and Tyr194, either directly or through a water molecule (w3 in Figure 6b,c,e,f). Moreover, the sulfonamide moiety may displace a water molecule (w2, shown in Figure 6e), occupying the same position observed in *Tb*PTR1. In most cases, the diamino-pyridinium moiety is oriented as the glutamate tail in either PDB IDs 1E7W or 2BFA (Figure 6b,e,f), establishing polar interactions with Tyr283, Arg287 and Ala288. Notably, the orientation of the Asp232 side chain (Pro210 in *Tb*PTR1) may drastically change the binding pose of **TCMDC-143249**, as reported in Figure 6c, showing that the diamino-pyridinium moiety might also be oriented to H-bond Asp232.

The different interactions made by **TCMDC-143249** in *Lm*PTR1 with respect to *Tb*PTR1 can be explained by the difference in the protein binding sites. Indeed, despite the overall conservation of the secondary structure, the two enzymes share 51% sequence identity and present some structural adjustments at the level of the binding site loop (Figure S3). The observed differences do not change the inhibition potency of the compound, showing IC_50_ of 13.5 and 6.0 μM against *Tb*PTR1 and *Lm*PTR1, respectively. Such variations change the local hydrophobic/polar interaction pattern and should be considered when targeting both *Tb*PTR1 and *Lm*PTR1. *Tb*PTR1 presents residues Glu217, Cys168 and Phe171, which correspond to Val237, Leu188 and Tyr191 in *Lm*PTR1, respectively. Moreover, the Arg287 side chain of the adjacent protomer C-terminus protrudes in *Lm*PTR1 active site (differently to His267 in *Tb*PTR1). An additional change includes the pABA (p-amino benzoic acid) binding site, flanked by Asp232 and His241 in *Lm*PTR1 (Pro210 and Trp221, respectively, in *Tb*PTR1). Asp232 in *Lm*PTR1 and Pro210 in *Tb*PTR1 belong to the substrate binding loop, whose conformation and residue composition may affect ligand binding. The different primary sequence of this loop (residues 207–215 in *Tb*PTR1, and residues 230–238 in *Lm*PTR1) may explain the differential activity of some ligands between the two PTR1 enzymes. The increased flexibility of the substrate binding loop in *Lm*PTR1 with respect to *Tb*PTR1 is a double-edged sword, giving the advantage of adding a bulkier substituent for improving binding affinity, and the disadvantage of its dynamic unpredictability in docking studies. To account for the substrate loop flexibility in our docking studies, we used several different *Lm* and *Tb*PTR1 X-ray structures (Table S1). We considered, in particular, protein structures co-crystallized with folate-like, antifolate-like and antifolate-like with bulkier substituent molecules, also taking into account the structure resolution and completeness. In this way, we were able to consider different conformation of the substrate-loop, the only flexible region of the binding site.

**Figure 7:**
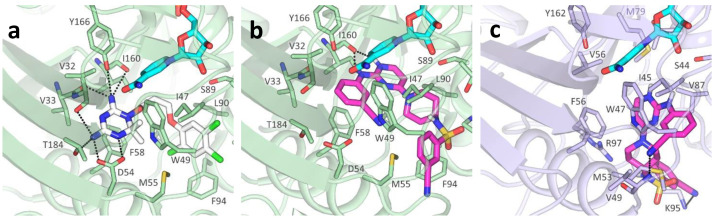
**TCMDC-143249** docking poses in *Tb* and *Lm*DHFR (**a**). Pyrimethamine inhibitor (white) main polar contacts in PDB ID 3RG9. Docking pose of **TCMDC-143249** (magenta) in *Tb*DHFR (**b**), and in *Lm*DHFR model (**c**). Protein is represented as cartoon (*Tb*DHFR, light green; *Lm*DHFR, violet), with relevant binding site residues depicted as sticks and labelled. NADPH cofactor (cyan) and ligands are shown as capped sticks. For clarity, polar hydrogens are shown for ligands only.

Docking studies in DHFR-TS show that **TCMDC-143249** does not fulfill active site requirements, particularly in the pteridine subsite. In *Tb*DHFR (PDB ID 3RG9), despite the π–π interaction with Phe58 and the nicotinamide ring, **TCMDC-143249** does not trace any of the acceptor/donor features found in pyrimethamine (PYR) inhibitors (Figure 7a,b). Important interactions with Asp54, Tyr166, Thr184 and the backbone of Val32, Val33 and Ile160 were never recorded in any pose for **TCMDC-143249**, and only an interaction with the backbone of Ile160 was observed. In particular, the sulfonamide group might hardly be stabilized by the hydrophobic environment created by Pro91, Leu90, Phe94, Leu97, Phe58 and Met55. Similarly, to what was reported for *Tb*DHFR-TS, docking of **TCMDC-143249** in the *Lm*DHFR-TS model highlighted no relevant key polar contact or hydrophobic interaction (Figure 7c). Even if the sulfonamide moiety may establish polar interactions with the Lys57 side chain and with the backbone of Met43, the cyano-phenyl diaminopyrimidine core misses the donor/acceptor requirements that stabilize the pteridine substrate. These findings point towards a likely instability of **TCMDC-143249** in *Tb*- and *Lm*DHFR-TS, hence providing a structural basis for the differential activity of **TCMDC-143249** in PTR1 and in DHFR-TS enzymes.

The other compounds indicated in Table 4 provide less effective inhibition and mainly lose the pan-inhibitor profile. **TCMDC-143191** shows an interesting activity only towards *Tb*PTR1 and assumes an orientation different from both the antifolate- and substrate-like ones, in which the pyrimidine nitrogen H-bonds Tyr174 and the ribose, the tricyclic system forms a hydrophobic interaction with Trp221 and the carbonyl contacts Cys168 (Figure S4a). Compound **TCMDC-143459** behaves similarly, showing an effect only towards *Tb*PTR1 and being able to profitably locate only in PDB ID 4CLO, where it H-binds to NADPH ribose and phosphates through the triazole and imidazole rings, and it forms a π–π sandwich with the cofactor and Phe97, and an additional π–π stacking with Trp204 through the terminal benzyl ring (Figure S4b). Compounds **TCMDC-143518** and **TCMDC-143386** present, on the contrary, better inhibition towards *Lm*PTR1 than *Tb*PTR1. **TCMDC-143518** difficultly fits in both PTR1 binding sites and finds a suitable pose only in the *Lm* enzyme, in PDB IDs 2BFA and 1W0C. Here, the standard connections with the cofactor and Tyr194 are mainly lost, apart from the weak H-bonds that can be formed by acidic pyrimidine hydrogens. However, the pyrimidine still forms a π–π sandwich with the cofactor and Phe113, one of the two pyrimidine nitrogen becomes closer to Arg17, the protonated amine interacts with the cofactor and a possible contact is formed by the benzimidazole with Arg287 (Figure S4c). **TCMDC-143386** assumes quite different poses according to the protonation state and to the X-ray structure of the protein. A particularly interesting pose of the compound is generated in *Lm*PTR1 (PDB ID 2BFA) and shown in Figure S4d. H-bonds are formed by the cyclic amide with Arg17 and the cofactor phosphate, and by the aniline nitrogen with the cofactor nicotinamide. The π–π sandwich is maintained, and an additional H-bond is formed by the terminal hydroxyl with Tyr283. Hydrophobic contacts are formed with Tyr191, Leu229 and Val230.

## 3. Materials and Methods

### 3.1. Reagents

BH2 (7,8-dihydro-L-biopterin) 37272, NADPH (β-nicotinamide adenine dinucleotide 2′-phosphate reduced tetrasodium salt hydrate) A1395, Cyt C (Cytochrome C) C2037, sodium citrate buffer S4641/C1909, DMSO 472301, TES (N- [Tris(hydroxymethyl)methyl]-2-aminoethanesulfonic acid) T1375, MgCl_2_ (Magnesium Chloride hexahydrate) M9272, β-Me (2-Mercaptoethanol) M3148, DHF (7,8-Dihydropteroyl-L-glutamic acid) D7006 and MTX (Methotrexate) A6770 were purchased from Merck. Round bottom clear polystyrene Corning^®^ NB.15 96-well plates were purchased from Merck (CLS3798-100EA).

### 3.2. In Silico Chemoinformatic and Clustering Analysis

The structural features and drug-likeness properties of the GSK Kinetobox collection were calculated in silico by using QikProp tool (Maestro Schrödinger, New York, NY, USA) [35]. A single binary *2D* fingerprint was also calculated for each chemical compound, considering an extended connectivity fingerprinting 4-ECFP4, in which the atoms and the bonds were distinguished by functional type and hybridization, respectively. Next, a similarity–distance matrix was obtained based on Tanimoto coefficient (=0.85), which was used for performing a hierarchical clustering (bottom-up approach) using the complete clustering linkage as an agglomerative clustering method. The same similarity matrix was also used as input data for RStudio open-source software (https://rstudio.com/, accessed on 13 October 2020) [36] to visually represent, as a dendrogram, the chemical similarities between molecules. We used the hclust statistical function available on the software tool and then translated the resulting clustering matrix (*csv* file) to tree file format, which was finally used as input for the iTOL online server (http://itol.embl.de/, accessed on 9 November 2020) [37] for displaying the circular cladogram shown in Figure 1.

### 3.3. Protein Purification

*Lm/Tb*PTR1 and *Lm/Tb*DHFR-TS genes were cloned in pET15b vectors. A modified vector, having the canonical thrombin recognition site replaced by that of TEV protease (pET15b-TEV vector), was used for *Lm*DHFR-TS gene cloning. *Lm/Tb*PTR1 were produced in *E. coli* BL21(DE3) as His-tag proteins and purified by immobilized metal affinity chromatography (IMAC), as formerly reported by Borsari et al. [38], with minor modifications. Briefly, bacterial cells were cultured at 37 °C in SuperBroth (SB) media (including 100 mg/L ampicillin) to mid-log phase and the target over-expression was induced with 1 mM isopropyl-β-D-thiogalactopyranoside (IPTG) overnight at 24 °C, for *Tb*PTR1 and with 0.4 mM IPTG overnight at 28 °C for *Lm*PTR1. Cells, harvested by centrifuge, were resuspended in 50 mM Tris-HCl, pH 7.5, 250 mM NaCl and 20 mM imidazole, and disrupted by sonication. The supernatants of the resulting crude extracts were collected by centrifuge and loaded on a HisTrap FF 5 mL column (GE Healthcare). *Lm/Tb*PTR1 were purified using a three-step gradient protocol by applying an imidazole concentration of 250 mM in the same buffer. The resulting protein samples were combined with thrombin protease (3 units/mg target protein) and then dialyzed overnight at 8 °C in 50 mM Tris-HCl, pH 7.5, 0.25 M NaCl and in 50 mM Tris-HCl, pH 7.5, for *Tb*PTR1 and *Lm*PTR1, respectively (membrane cutoff 10 kDa). The mature *Lm/Tb*PTR1 were further purified through a second IMAC stage, where they were eluted as weakly bound proteins, by applying an imidazole concentration of 10–50 mM (in the same buffers). The purified proteins were dialyzed overnight at 8 °C in 50 mM Tris-HCl, pH 7.5, 0.25 M NaCl and in 50 mM Tris-HCl, pH 7.5, for *Tb*PTR1 and *Lm*PTR1, respectively, and stored at −80 °C added by 10–20% glycerol.

*Lm/Tb*DHFR-TS were produced in *E. coli Artic-Express* (DE3) as His-tag proteins and purified by IMAC as formerly reported, with minor modifications [38]. Briefly, bacterial cells were cultured in ZYP5052 autoinduction media (supplemented with 100 mg/L ampicillin) at 30 °C to OD_600nm_ values of ~1.0 and then incubated at 12 °C for 60–72 h under vigorous aeration [39]. Cells, harvested by centrifuge, were resuspended in 50 mM sodium citrate, pH 5.5, 250 mM NaCl, and in 50 mM Tris-HCl, pH 8, 250 mM NaCl, 10% glycerol, for *Lm*DHFR-TS and *Tb*DHFR-TS, respectively, and then disrupted by sonication. The supernatants of the resulting crude extracts (collected by centrifuge) were loaded on a HisTrap FF 5 mL column (GE Healthcare) and purified using a three-step gradient protocol by applying an imidazole concentration of 200–400 mM (in the same buffers). The resulting sample of *Lm*DHFR-TS was combined with TEV protease (0.05–0.1 mg TEV/mg target protein) and then dialyzed overnight at 8 °C in 50 mM sodium citrate, pH 5.5, 250 mM NaCl (membrane cutoff 10 kDa). On the other hand, the sample of *Tb*DHFR-TS was combined with thrombin protease (3 units/mg target protein) and dialyzed overnight at 8 °C in 50 mM Tris-HCl, pH 8, 250 mM NaCl, 10% glycerol. Mature *Lm/Tb*DHFR-TS were subjected to a second IMAC stage, where they were collected as unbound proteins. The resulting samples of the mature *Lm/Tb*DHFR-TS were further purified by size-exclusion chromatography on a HiLoad 16/600 Superdex 200pg column (GE Healthcare) equilibrated with the respective buffers. The purified proteins were dialyzed overnight at 8 °C in 50 mM sodium citrate, pH 5.5, 250 mM NaCl and in 50 mM Tris-HCl, pH 8, 250 mM NaCl, for *Lm*DHFR-TS and *Tb*DHFR-TS, respectively, and stored at −80 °C added by 10–20% glycerol. The high purity of all purified proteins was confirmed by SDS-PAGE analysis and MALDI-TOF mass spectrometry. The final protein yields were assessed as approximately 60 mg/L and 45 mg/L bacterial culture, for *Tb*PTR1 and *Lm*PTR1, respectively, and as approximately 10 mg/L for *Lm/Tb*DHFR-TS.

### 3.4. Anti-Kinetoplastid Chemical Boxes

The HAT, CHAGAS and LEISH chemical boxes were provided by GlaxoSmithKline. The collection comprised 592 compounds, prepared as 10 mM stock solutions in DMSO (10 μL each) and dispensed in 384-well plates. For primary screening, a working solution (final concentration of 2 mM) for each compound was prepared in 96-well plates by 1:5 dilution in DMSO while 1 μL of the 10 mM stock solution was used for secondary screening of selected compounds.

#### 3.4.1. Primary Screening

Kinetobox collection was tested on the recombinant *Lm/Tb*PTR1 protein by a Cytochrome C (Cyt-C) coupled-spectrophotometric assay with a 96-well multiplate reader (Spectramax-190, Molecular Device) [40,41]. Each compound was properly diluted to have a final concentration of 10 µM and a DMSO percentage ≤ 1% in the enzyme mixture. Methotrexate (MTX) was included into the screening panel as *C^+^* control at final concentration of 1 µM (IC50 equal to 1 µM and 0.5 µM for *Tb*- and *Lm*-PTR1, respectively) [42]. Then, 1 μL of each diluted compound stock (2 mM in DMSO) was manually added to the plate (in triplicate). The first and the last row of each plate were used for C^+^ (MTX) and C^−^ (no-inhibition) controls to reduce any positional and/or association bias. This step was followed by the addition of 100 μL of 20 mM sodium citrate pH 6.0, 80 μM Cyt-C, 3 μM and 0.3 μM BH2 (for *Lm* and *Tb*, respectively), 0.002 μM and 0.02 μM (for *Lm* and *Tb*PTR1, respectively) and double-distilled water (0.2 µm filtered) to volume. After homogenization, 10 min of incubation at 30 °C and shaking for 1 min, 100 μL of activity buffer containing NADPH (500 μM) and sodium citrate 20 mM was added to each well. After brief shaking, the reading was performed for a total kinetic time of 10 min at 30 °C at 550 nm. Raw screening measurements were used to determine the slope of progression curves by linear regression for control and compound wells. The percent inhibition (%*Inh*) was calculated for each compound as follows: %*Inh* = 100 − [(dOD/dt)^well^ * 100]/µ^C−^, where (dOD/dt)^well^ represents the slope of each compound well and µ^C−^ the average of no-inhibition controls [24]. The data results are the mean of two experiments performed in triplicate.

#### 3.4.2. Secondary Screening (Dose–Response Curve)

Fourteen compounds selected from primary screening were tested on *Lm/Tb*DHFR-TS recombinant protein in a dose–response manner (final concentration ranging from 40 μM to 10 μM) by a spectrophotometric assay monitoring the enzyme kinetics of reduction reaction of DHF substrate to THF, at λ = 340 nm for 180 s [43,44]. Then, 1 μL of each compound stock (10 mM in DMSO) was used to prepare diluted stocks (8, 4 and 2 mM) corresponding to the concentration points to assay (40, 20 and 10 μM). In this way, considering a final volume of 200 μL, it was possible, taking out 1 μL of compound from each diluted stock, to always guarantee a percent value of DMSO ≤1% in the reaction mixture. Each concentration was tested in triplicate and the resulting IC50s represented the mean of two experiments. Additionally, in this case, the known inhibitor MTX was used as positive control at a concentration of 0.002 μM (IC50 = 0.001 and 0.002 μM for *Lm* and *Tb* DHFR-TS, respectively) [45,46]. The first and the last rows of plates were used for C^+^ (MTX) and C^−^ (no-inhibition) controls to reduce any positional and/or association bias. After compound dispensing, 100 μL of TES buffer (TES 100 mM, MgCl_2_ 50 mM, β-ME 150 mM), 50 μM DHF substrate, DHFR-TS recombinant enzyme (0.022 µM and 0.086 µM for *T. brucei* and *L. major*, respectively) and double-distilled water (0.2 µm filtered) to volume were added to each well. After homogenization by shaking for 1 min, 100 μL of activity buffer containing 120 μM NADPH and TES buffer was added to the plate for starting the reaction. After brief shaking, the reading was performed for a total kinetic time of 180 s at room temperature at 340 nm. From the resulting inhibition percentages at each different inhibitor concentration, and assuming a competitive inhibition mechanism, it was possible to estimate the IC50 values by fitting the four-parameter Hill equation to experimental data from dose–response curves using the GraphPad Prism software [47].

### 3.5. Molecular Modelling

The protein structure of *Lm*DHFR-TS (Uniprot code: P07382) was modelled using SWISS-Model Protein Modelling Server (https://swissmodel.expasy.org/, accessed on 26 July 2020) [48]. PDB ID 3INV (*T. cruzi* DHFR) was chosen as the template structure, sharing 68.50% sequence identity with the target sequence. Quality of the homology model was assessed by the QMEAN scoring function (QMEAN = 0.9) provided by the SWISS-Model server and the NADPH cofactor was retained from the template structure (the model is available upon request to the authors; the Ramachandran plot is reported in Figure S5). Prior to docking studies, proteins were prepared using Sybyl version 7.0 software (http://www.tripos.com), adding hydrogens and keeping the PTR1 tetrameric and DHFR-TS dimeric biological assemblies. The selected 14 compounds were retrieved as SMILES code and translated with Open Babel [49]. Their tautomeric/protonation state at the tested pH (3–4) was checked using the MoKa software [50]. Compounds were submitted to docking with GOLD version 2.2 [51] using standard parameters. Genetic algorithm 50-runs were performed for each ligand to explore as many conformations as possible, and key water molecules were retained with the toggle option. Eventually, poses were scored with CHEMPLP function and ranked accordingly.

## 4. Conclusions

**TCMDC-143249**, belonging to the LEISH Kinetobox, is the most interesting molecule showing a benzenesulfonamide structure, as defined by the QiqProp descriptor tool. It was selected by MTS approach showing a pan-inhibitors profile: it is a non-pteridine-like compound, inhibiting PTR1 from both parasitic agents *Leishmania* and *Trypanosoma* (IC_50_ values of 6.0 µM and 13.5 µM, respectively), with no inhibition of *Lm* or *Tb*DHFR-TS enzymes. It can inhibit the growth of all three kinetoplastidic parasites, *L.donovani*, *T.brucei* and *T.cruzi*. Despite the fact that benzenesulfonamide compounds are well known among antimicrobial agents, this is not a largely explored core structure in anti-kinetoplastidic parasitic infections.

Molecular modelling studies show that **TCMDC-143249** binds the active site of *Lm* and *Tb*PTR1 but does not fulfill the active site requirements for the binding to *Lm/Tb*DHFR-TS enzyme, pointing towards a likely instability in the complex with *Tb* and *Lm*DHFR-TS. This provides a structural basis for the differential activity of **TCMDC-143249** in PTR1 and in DHFR-TS enzymes, in agreement with the experimental data. This molecule could thus represent a promising template for further design and development of new inhibitors by mimicking the same pattern of interactions with the target enzymes.

Further development of the medicinal chemistry program will require the re-synthesis and an SAR-based library design around the **TCMDC-143249** compound. Its typical modular structure with four main fragments (cianobenzene, pyrimidine, piperidine and a benzenesulfonamide ring) can be decorated in all fragments independently. To speed up the process, we already have a docking model of the compound with all enzymes studied ready for computational studies. An X-ray structure of the complex of **TCMDC-143249** with *Lm*PTR1 and *Tb*PTR1 can be obtained and docking studies for optimized library design can be performed. Considering the molecular properties of the hit, such as pKa_s_ and logD, these should be carefully evaluated, because the electronic properties and overall molecular states will influence both the target interaction and the in vivo pharmacokinetic. Hit’s cLogP is 3.16; therefore, we will improve this feature by adding hydrophilic substituents to have a greater interaction with the solvent, aiming to make the compound suitable for oral administration and intestinal absorption (adequate bioavailability). The structural changes should not affect the compound’s binding mode or in vitro activity towards the target protein. An alternative and particularly attractive approach for improving aqueous solubility without an increase in molecular weight, which may have adverse consequences for the pharmacokinetics, can be also focused on more significant structural changes such as the disruption of molecular planarity and symmetry [52]. In conclusion, considering the need for new chemical entities to be included in the pre-clinical pipeline for Trypanosomiasis parasitic infections, this work may deliver improved treatments in the future.

## Figures and Tables

**Table 1:** Drug-likeness properties of the Kinetobox compound library.

Physicochemical Property	Minimum Value	Maximum Value	Average	Drug-Likeness Criteria	% Compounds According to RO5 *
MW (g/mol)	210	547	375	≤500	99.2%
AlogP	−2.7	5.8	2.8	≤5	98.7%
HBA	1	8	3.8	≤10	100%
HBD	0	5	1.3	≤5	100%
Total Polar Surface Area (Å^2^)	19	184	89.5	≤140	94.3%
N° of Rotatable Bonds	1	11	5.1	≤10	99.8%

*, % of compounds according to RO5 (cut-off selection: no more than one violation per compound) 92.2%.

**Table 2:** Pyrimido-pyrimidine derivatives (**cluster VIII**).

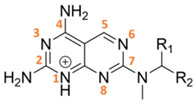									
		Substituents		IC_50_ (µM)				EC_50_ (µM)	
TCMDC ID	R_1_	R_2_	HTS_BOX	TbPTR1	LmPTR1	TbDHFR	LmDHFR	*T. brucei*	*L. donovani*
**143232**	H	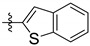	CHAGAS	10.9	5.9	-	24.4	28.4	29.5
**143295**	CH_3_	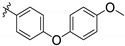	LEISH	7.9	6.7	-	16.6	N.D.	0.06
**143296**	CH_3_	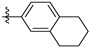	LEISH	8.6	6.5	9.7	6.9	14.2	1.8
**143297**	CH_3_	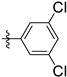	LEISH	9.8	5.7	11.6	20.1	35.7	0.6
**143298**	CH_3_	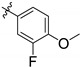	CHAGAS	8.1	6.0	-	-	8.8	10.8

No value (-) is reported when IC_50_ was higher than 40 µM. Standard errors are within ± 10% of the indicated value.

**Table 3:** Pyrido-pyrimidine (**I, cluster VI**), pyrrolo-pyrimidine (**II**) and pyrimidine (**III, cluster XI**) derivatives.

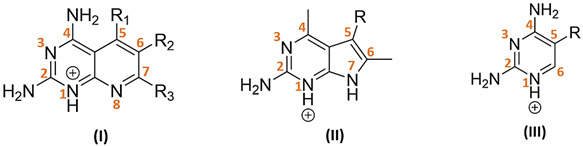										
I										
	Substituents				IC_50_ (µM)				EC_50_ (µM)	
TCMDC ID	R_1_	R_2_	R_3_	HTS_BOX	TbPTR1	LmPTR1	TbDHFR	LmDHFR	*T. brucei*	*L. donovani*
**143606** (VI)	H	CH_2_CH_3_	(CH_2_)_2_CH_3_	CHAGAS	6.4	6.8	-	19.4	22.5	27.2
**143607** (VI)	CH_3_	H	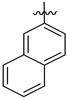	LEISH	7.3	8.1	-	-	25.8	2.7
**II**										
	**Substituents**				**IC_50_ (µM)**				**EC_50_ (µM)**	
**TCMDC ID**	**R**			**HTS_BOX**	**TbPTR1**	**LmPTR1**	**TbDHFR**	**LmDHFR**	***T. brucei***	***L. donovani***
**143610**	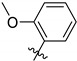			CHAGAS	7.3	9.3	38.2	40.0	32.5	17.9
**III**										
	**Substituents**				**IC_50_ (µM)**				**EC_50_ (µM)**	
**TCMDC ID**	**R**			**HTS_BOX**	**TbPTR1**	**LmPTR1**	**TbDHFR**	**LmDHFR**	***T. brucei***	***L. donovani***
**143611** (XI)	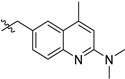			CHAGAS	8.9	9.8	-	-	5.0	-

No value (-) is reported when IC_50_ was higher than 40 µM. Standard errors are within ± 10% of the indicated value.

**Table 4:** Non-antifolate-like scaffolds. Core scaffolds reported in the cluster are highlighted in red boxes.

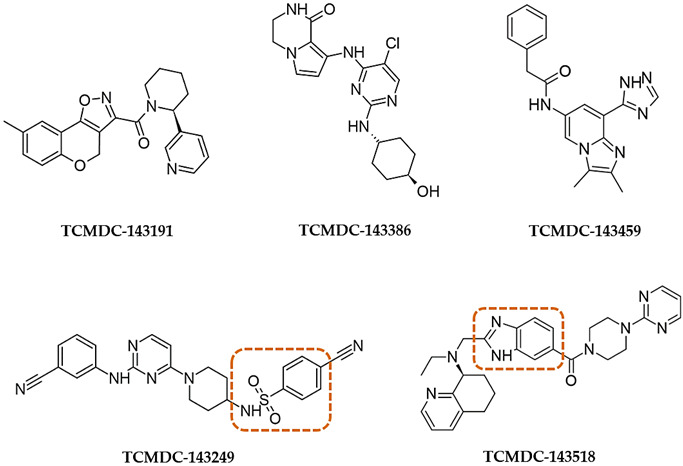							
		IC_50_ (µM)				EC_50_ (µM)	
TCMDC ID	HTS_BOX	TbPTR1	LmPTR1	TbDHFR	LmDHFR	*T. brucei*	*L. donovani*
**143191**	CHAGAS	9.8	38.5	-	-	39.8	-
**143249** (XVI)	LEISH	13.5	6.0	-	-	6.3	5.6
**143518** (X)	LEISH	33.3	8.5	-	25	3.8	3.5
**143386**	HAT	35.0	6.7	-	25.8	0.6	1.4
**143459**	LEISH	9.8	-	-	-	6.6	0.5

No value (-) is reported when IC_50_ was higher than 40 µM. Standard errors are within ± 10% of the indicated value.

## Data Availability

Data are contained within the article and Supplementary Materials.

## Supplementary Materials

The following are available online at https://www.mdpi.com/article/10.3390/ph14121246/s1. Content: Table S1 (references [14,41,53,54,55,56] are cited in the Supplementary Materials): Relevant information on target proteins retrieved from RCSB and used in docking studies. Figure S1: Antifolate- and substrate-like poses in PTR1 and in DHFR. Figure S2: Docking of the most relevant pyrido-pyrimidine, pyrrolo-pyrimidine and pyrimidine derivatives (Table 2) reported in Table 3 (Main Text). Figure S3: Comparison between LmPTR1 and TbPTR1 binding site, and details of the substrate binding loop. Figure S4: Docking pose of other compounds reported in Table 3 (Main Text). Figure S5: Ramachandran plot of the *Lm*DHFR-TS homology model. *Lm*DHFR-TS homology model available at model at FAIRDOM ID: https://fairdomhub.org/data_files/4313?version=1. accessed on 30 October 2021.
