# Supplementary material for: Repurposing the Trypanosomatidic GSK Kinetobox for the Inhibition of Parasitic Pteridine and Dihydrofolate Reductases
Source: Pharmaceuticals (Basel). 2021 Nov 30;14(12):1246. doi: 10.3390/ph14121246 (PMC8704748; doi:10.3390/ph14121246)
Supplement: Supplementary file 1 [file pharmaceuticals-14-01246-s001.zip › pharmaceuticals-1466007-supplementary.pdf]

pharmaceuticals

MDPI

Supplementary information

Repurposing the Trypanosomatidic GSK Kinetobox for the Inhibition of Parasitic Pteridine and Dihydrofolate Reductases

Matteo Santucci [1,†], Rosaria Luciani [1,†], Eleonora Gianquinto [2,†], Cecilia Pozzi [3], Flavio di Pisa [3], Lucia dello Iacono [3], Giacomo Landi [3], Lorenzo Tagliazucchi [1], Stefano Mangani [3], Francesca Spyrakis [2] and Maria Paola Costi [1,*]

[1] Department of Life Science, University of Modena and Reggio Emilia, Via Campi 103, 41125 Modena, Italy; matteo.santucci86@gmail.com (M.S.); rosaria.luciani@libero.it (R.L.); lorenzo.tagliazucchi@unimore.it (L.T.)
[2] Department of Drug Science and Technology, University of Turin, Via Giuria 9, 10125 Turin, Italy; eleonora.gianquinto@unito.it (E.G.); francesca.spyrakis@unito.it (F.S.);
[3] Department of Biotechnology, Chemistry and Pharmacy—Department of Excellence 2018–2020, University of Siena, Via Aldo Moro 2, 53100 Siena, Italy; pozzi4@unisi.it (C.P.); dipisa2@unisi.it (F.d.P.); delloiacono3@unisi.it (L.d.I.); landi31@unisi.it (G.L.) stefano.mangani@unisi.it (S.M.)
* Correspondence: mariapaola.costi@unimore.it
† These Authors contribute equally to the work.

Citation: Santucci, M.; Luciani, R.; Gianquinto, E.; Pozzi, C.; Pisa, F.d.; Iacono, L.d.; Landi, G.; Tagliazucchi, L.; Mangani, S.; Spyrakis, F.; et al. Repurposing the Trypanosomatidic GSK Kinetobox for the Inhibition of Parasitic Pteridine and Dihydrofolate Reductases. *Pharmaceuticals* **2021**, *14*, 1246. https://doi.org/10.3390/ph14121246

Academic Editor: Christophe Dardonville

Received: 31 October 2021
Accepted: 25 November 2021
Published: 30 November 2021

Publisher's Note: MDPI stays neutral with regard to jurisdictional claims in published maps and institutional affiliations.

Copyright: © 2021 by the authors. Licensee MDPI, Basel, Switzerland. This article is an open access article distributed under the terms and conditions of the Creative Commons Attribution (CC BY) license (https://creativecommons.org/licenses/by/4.0/).

Table S1. Relevant information on target proteins retrieved from RCSB and used in docking studies. Docking studies on PTR1 and DHFR were carried out using several X-ray structures and a homology model. PDB IDs were chosen prioritizing bulky and diverse co-crystallized ligands when possible.

Target protein	PDB ID	Res. (Å)	Ligand Structure
***Tb*PTR1**	2C7V [41]	2.20	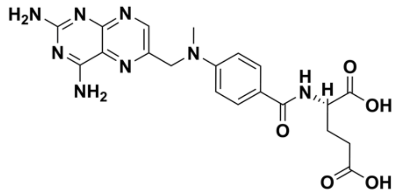
	2X9G [41]	1.10	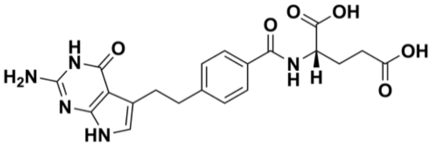
	4CLO [53]	1.88	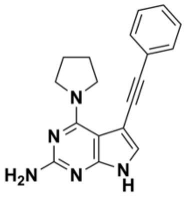
***Lm*PTR1**	2BFA [54]	2.70	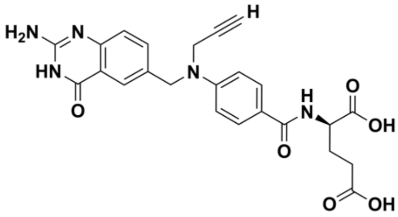
	1E7W [14]	1.75	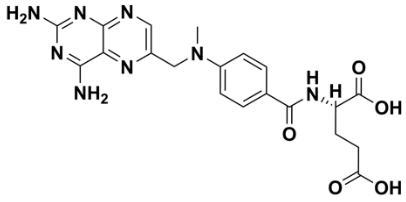
	1W0C [55]	2.60	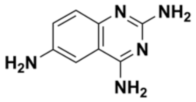
***Tb*DHFR**	3RG9 [56]	2.00	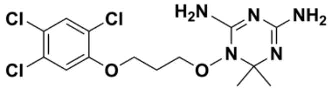

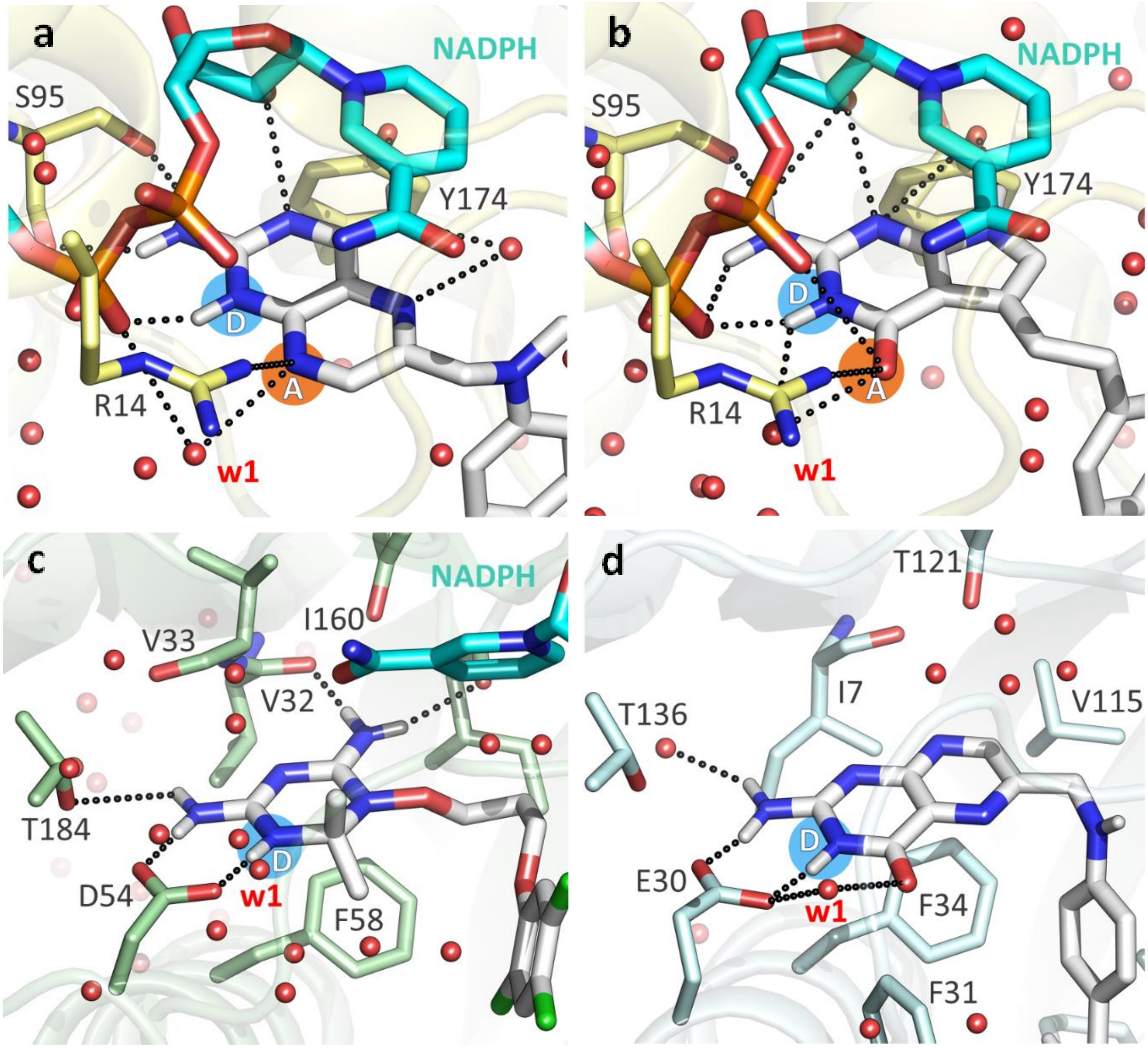

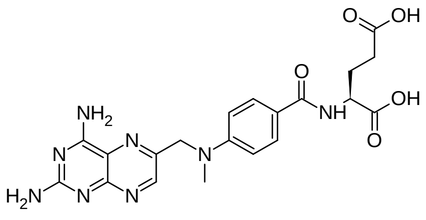

(**e**) Methotrexate

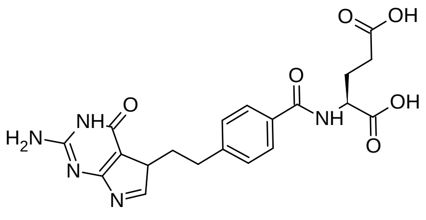

(**f**) Raltitrexed

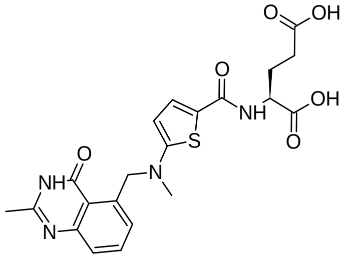

(**g**) Pemetrexed

Figure S1. Antifolate- and substrate-like poses in PTR1 and in DHFR. **a.** Antifolate-like pose in *Tb*PTR1 (PDB ID:2C7V, *Tb*PTR1 complexed with MTX). **b.** Substrate-like pose in *Tb*PTR1 (PDB ID:2X9G, *Tb*PTR1 complexed with pemetrexed). **c.** Antifolate-like pose in *Tb*DHFR (PDB ID:3RG9, *Tb*DHFR complexed with WR99210). **d.** Substrate-like pose in human DHFR (PDB ID:1DHF, human DHFR complexed with folate, missing NADPH cofactor). No x-ray structure of *Lm*DHFR-TS is currently available, thus, human DHFR has been used instead to present the substrate-like pose. Co-crystallized ligand (white), NADPH cofactor (cyan) and binding site residues (*Tb*DHFR-TS, light green; human DHFR, light blue; *Tb*PTR1, light yellow are depicted as sticks, and labelled. Water molecules are shown as red spheres. A relevant water

molecule for the ligand-protein or ligand-NADPH interaction is labelled as 'w1'. Key H-bond donor or acceptor sites on the ligand are enclosed in a light blue or orange circle and labelled '*D*' or '*A*', respectively Below are represented the chemical structures of (**e**) Methotrexate (MTX), (**f**) raltitrexed (RTX), and (**g**) pemetrexed.

.

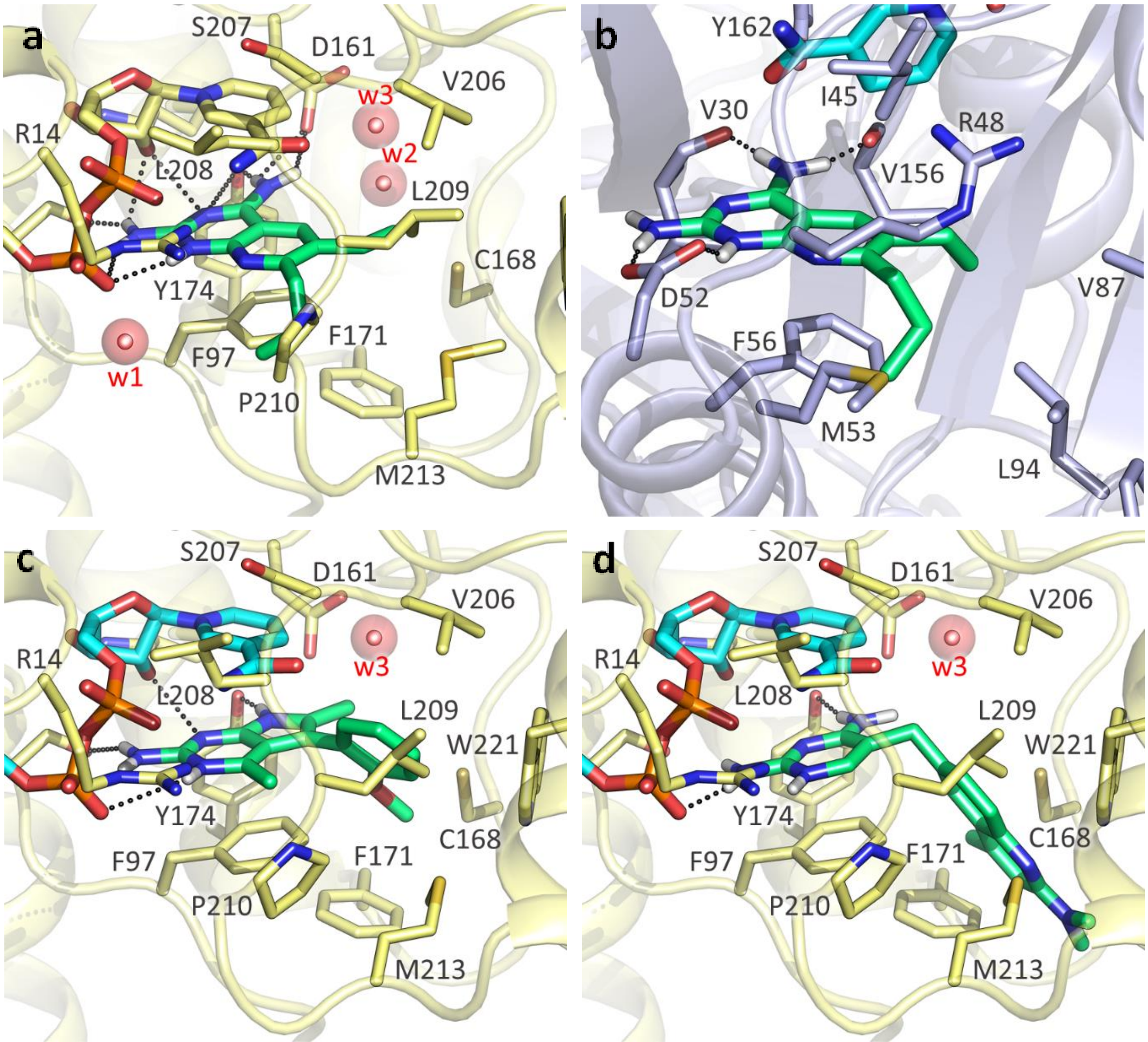

Figure S2. Docking of the most relevant pyrido-pyrimidine, pyrrolo-pyrimidine and pyrimidine derivatives (Table 3, Main Text). **a.** TCMDC-143606 in *Tb*PTR1 (PDB ID 4clo). **b.** TCMDC-143606 in *Lm*DHFR-TS. **c.** TCMDC-143610 in *Tb*PTR1 (PDB ID 2C7V). **d.** TCMDC-143611 in *Tb*PTR1 (PDB ID 2C7V). Waters are indicated when not displaced by the docking.

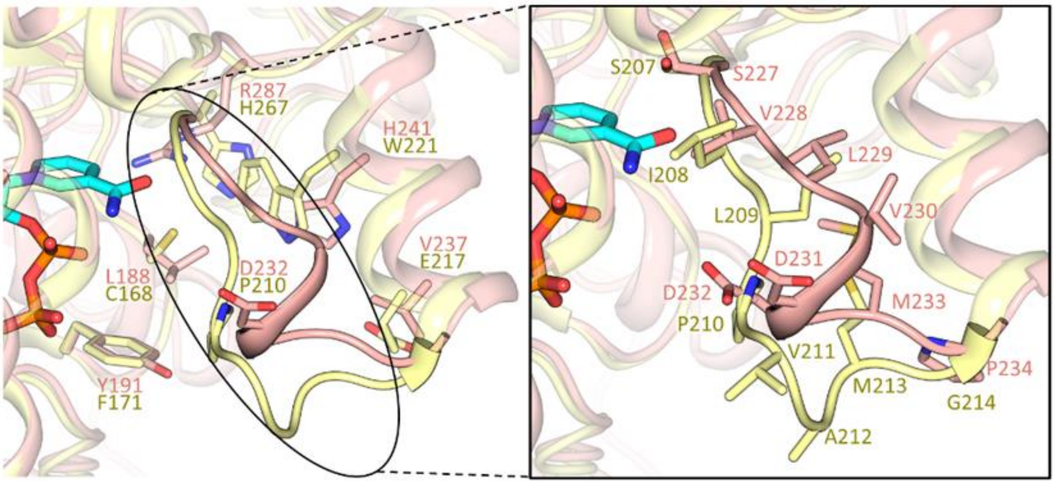

Figure S3. Comparison between *Lm*PTR1 and *Tb*PTR1 binding site, and details of the substrate binding loop. Protein is represented as cartoon (*Tb*PTR1, light yellow, PDB ID 2C7V; *Lm*PTR1, pink, PDB ID 1E7W), with relevant binding site residues depicted as sticks and labelled, and NADPH cofactor (cyan) as sticks. For clarity, hydrogens and water molecules are omitted.

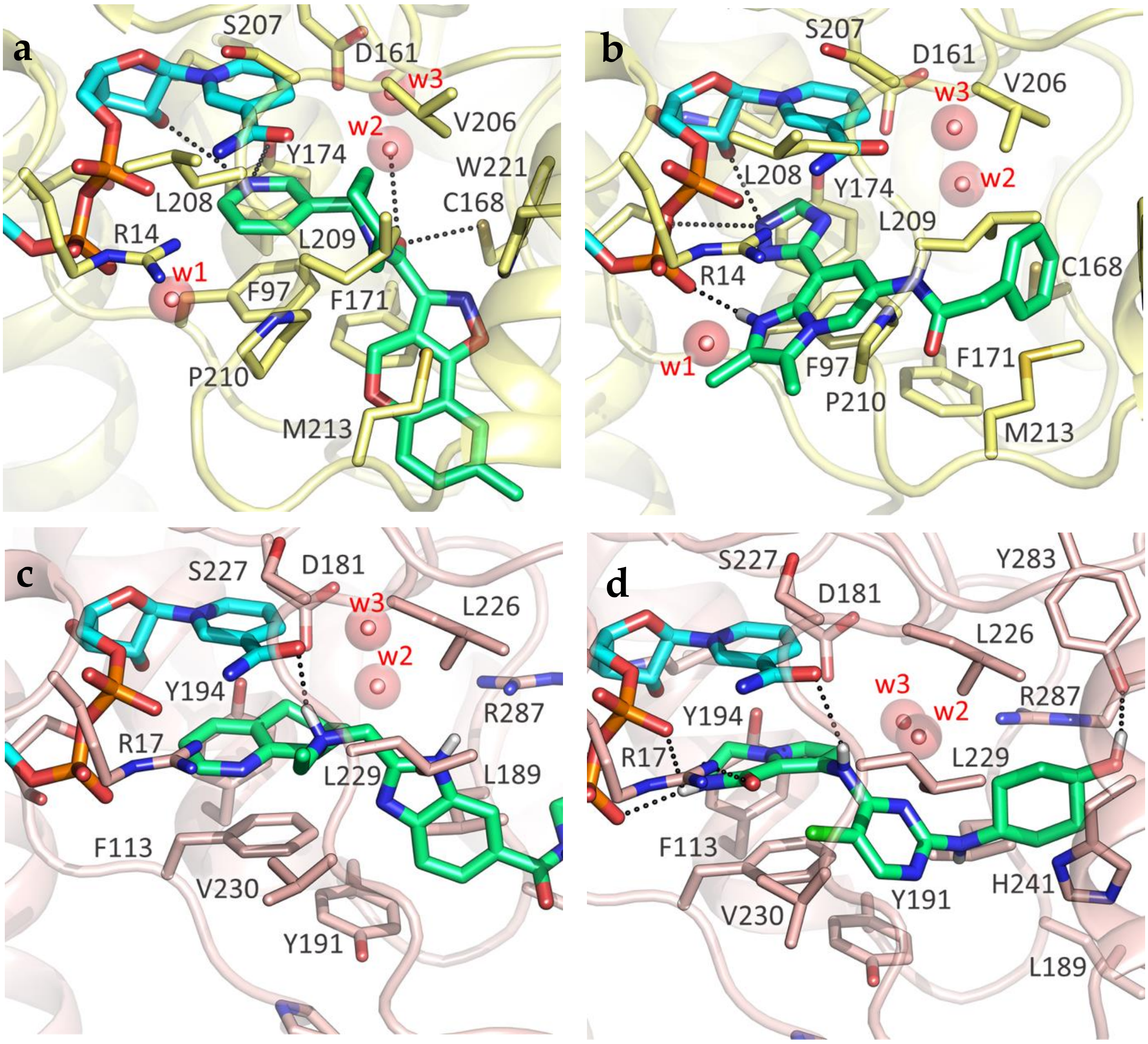

Figure S4. Docking pose of other compounds reported in Table 4 (Main Text). **a.** TCMDC-143191 in *Tb*PTR1 (PDB ID 2X9G). **b. TCMDC-143459** in *Tb*PTR1 (PDB ID 4CLO). **c. TCMDC-143518** in *Lm*PTR1 (PDB ID 1W0C). **d**. **TCMDC-143386** in *Lm*PTR1 (PDB ID 2BFA). Protein is represented as cartoon (*Tb*PTR1, light yellow; *Lm*PTR1, pink), with relevant binding site residues depicted as sticks and labelled. NADPH cofactor (cyan) and ligands are shown as capped sticks, polar interactions between ligands and protein are shown as black dashed lines. Water molecules are, indicated when not displaced by the docking. For clarity, polar hydrogens are shown for ligands only.

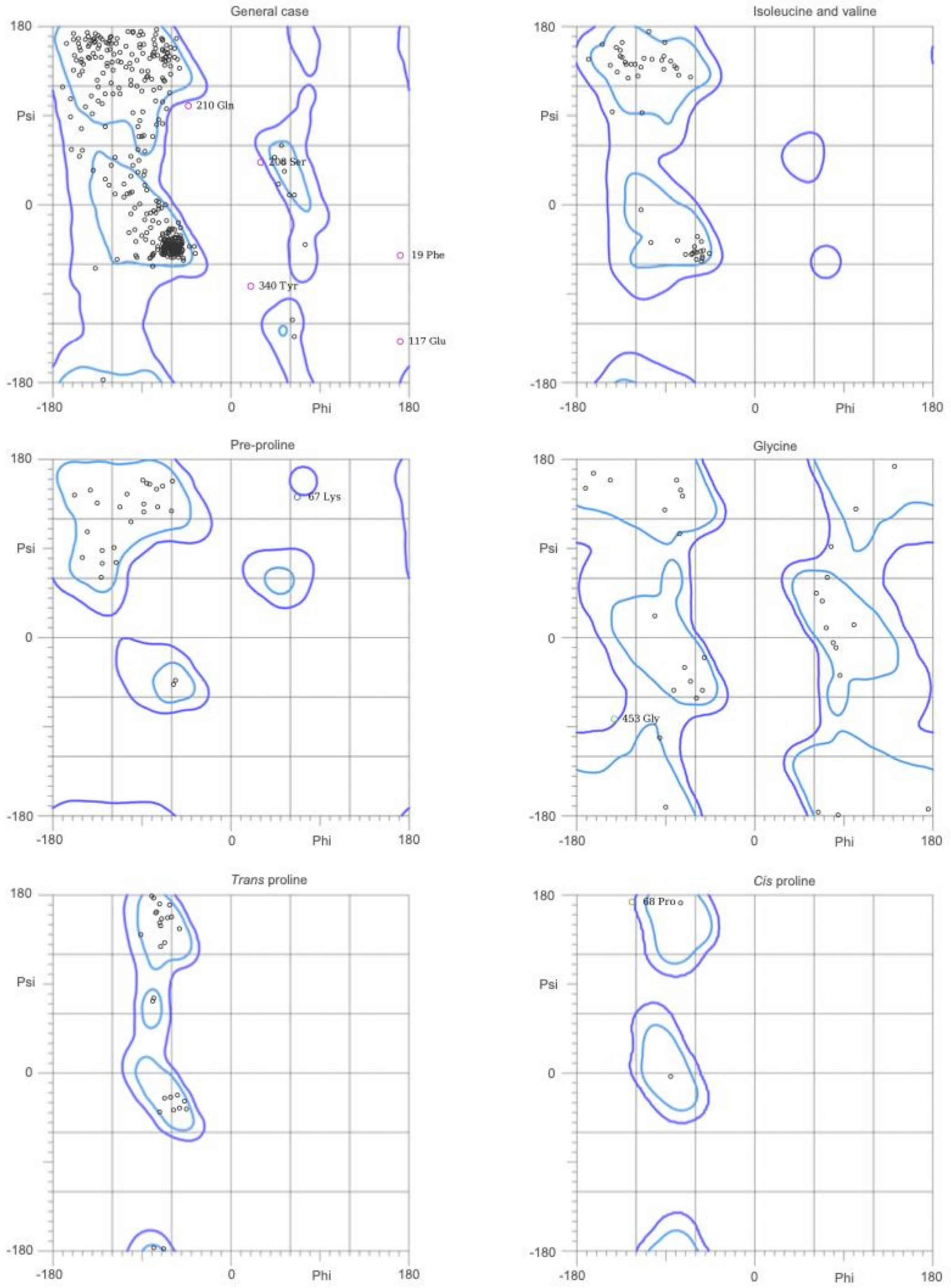

92.1% (465/505) of all residues were in favored (98%) regions.
98.4% (497/505) of all residues were in allowed (>99.8%) regions.

There were 8 outliers (phi, psi):
19 Phe (171.3, -51.7)
67 Lys (67.5, 143.0)
68 Pro (-124.7, 173.6)
117 Glu (171.2, -138.1)
208 Ser (30.4, 43.5)
210 Gln (-43.2, 100.3)
340 Tyr (20.7, -82.9)
453 Gly (-142.0, -82.2)

Figure S5. Ramachandran plots of the LmDHFR-TS model.
